# Schistosomiasis control in Senegal: results from community data analysis for optimizing preventive chemotherapy intervention with praziquantel

**DOI:** 10.1186/s40249-023-01155-3

**Published:** 2023-11-27

**Authors:** Boubacar Diop, Khadime Sylla, NDèye MBacké Kane, Oumou Kaltoum Boh, Babacar Guèye, Mady Ba, Idrissa Talla, Malang Mané, Rose Monteil, Boniface Kinvi, Honorat Gustave Marie Zoure, Jorge Cano Ortega, Pauline Mwinzi, Moussa Sacko, Babacar Faye

**Affiliations:** 1 Programme National de Lutte Contre les Bilharzioses et les Géo-Helminthiases, Direction de la Lutte Contre la Maladie (DLM), Ministère de la Santé et de l’Action Sociale (MSAS), Dakar, Senegal; 2 Department of Parasitology and Mycology Faculty of Medicine, Pharmacy and Odontology, Université Cheikh Anta Diop de Dakar (UCAD), Dakar, Senegal; 3 WHO Country Office, Dakar, Senegal; 4 Département de Santé Publique, Faculté des Sciences de la Santé, Université Amadou Hampathé Ba, Dakar, Senegal; 5 FHI 360, Chargé des Enquêtes et Evaluation des Maladies et des Campagnes de DMM, Dakar, Senegal; 6 Expanded Special Project for Elimination of Neglected Tropical Diseases (ESPEN), WHO Africa Regional Office, Brazzaville, Congo; 7 Service de Parasitologie, Institut National de Santé Publique (INSP), BP 1771 Bamako, Mali

**Keywords:** Schistosomiasis, Community data analysis, Endemicity, Praziquantel, Senegal

## Abstract

**Background:**

Over the past two decades, preventive chemotherapy (PC) with praziquantel (PZQ) is the major strategy for controlling schistosomiasis in Senegal. The objective of this analysis was to update the endemicity of schistosomiasis at community level for better targeting mass treatment with PZQ in Senegal.

**Methods:**

Demographic and epidemiological data from 1610 community health areas were analyzed using the schistosomiasis community data analysis tool of Expanded Special Project for Elimination of Neglected Tropical Diseases which developed by World Health Organization/Africa Office (WHO/AFRO). The tool uses a WHO/AFRO decision tree for areas without epidemiological data to determine whether mass treatment should be continued at community level. Descriptive analysis was performed.

**Results:**

Overall, the endemicity of 1610 community health areas were updated based on the data from the district endemicity (33.5%) and the form of Join request for selected PC medicine (40.5%). Up to 282 (17.5%) and 398 (24.7%) of community health areas were classified as moderate and high endemicity. 41.1% of communities were non endemic. High endemicity was more important in Tambacounda, Saint Louis, Matam, Louga and Kedougou. A change in endemicity category was observed when data was disagregted from district level to community level. Implementation units classified non endemic were more important at community level (*n* = 666) compared to district level (*n* = 324). Among 540 areas previously classified high endemic at district level, 392 (72.6%) remained high prevalence category, while 92 (17.0%) became moderate, 43 (8.0%) low and 13 (2.4%) non-endemics at community level. Number of implementation units requiring PC was more important at district level (1286) compared to community level (944). Number of school aged children requiring treatment was also more important at district level compared to community level.

**Conclusions:**

The analysis to disaggregate data from district level to community level using the WHO/AFRO schistosomiasis sub-district data optimization tool provide an update of schistosomiasis endemicity at community level. This study has allowed to better target schistosomiasis interventions, optimize use of available PZQ and exposed data gaps.

## Background

Schistosomiasis is a major waterborne neglected tropical disease caused by parasitic trematodes of the *Schistosoma* genus. The disease remains major public health problem in several part of the world, being most prevalent in developing countries where environmental and social factors contribute to the development of the disease. According to World Health Organization (WHO) more than 220 million people are annually estimated to be infected, mostly in sub-Saharan Africa [1, 2].

In Senegal, schistosomiasis became a major public health problem following the construction of the Diama Dam in Richard Toll 1988. The epidemiology of the disease along the Senegal River Basin has been dramatically changed with frequent outbreaks of intestinal and urogenital schistosomiasis [3, 4]. Schistosomiasis exists in two forms: (i) intestinal due to *S. mansoni*, mainly focused on the river valley, Kolda and Kédougou, located respectively in the north, south and south-east of the country and (ii) and the urinary or urogenital form due to *S. haematobium* present in all regions except Dakar [5–7].

In 1995, a Control Programme funded by European Union was set up in Saint-Louis region, following the increase of intestinal bilharziasis cases, around the Diama Dam at the mouth of the Senegal River. In 1997, with World Bank support, the Ministry of Health (MoH) has launched a control program for urinary schistosomiasis [8].

To fight against the disease, the MoH has implemented a National Schistosomiasis Control Programme in Senegal (Programme National de Lutte contre la Bilharziose, PNLB) in 1999 with the objective of reducing the morbidity related to urogenital and intestinal schistosomiasis by regular and appropriate mass drug administration (MDA) with praziquantel (provided by WHO) for school-aged children (SAC) [8].

National statistics in Senegal suggest that prevalence of urogenital schistosomiasis (caused by *S. haematobium*) ranges from 10% in the central regions, where transmission is seasonal, to more than 95% in the Senegal River basin, where transmission is perennial. Prevalence of intestinal schistosomiasis (caused by *S. mansoni*) was low in the central regions but in the Senegal Basin River (Dagana, Richard Toll) it can reach or even exceed 80% [9, 10].

To respond to the regional and global initiative launched by WHO, the MoH has developed and implemented two strategic plans for the periods 2007–2011 and 2011–2016. These two plans were essentially devoted to all neglected tropical diseases (NTDs) with a focus on preventive chemotherapy NTDs. The objective was to achieve the mapping of all endemics NTDs in Senegal.

The first disease mapping was conducted between 1996 and 2003 by the MoH. During this period, 102 villages in 29 districts have been mapped. Between 2009 and 2010, the PNLB, with the support from partners continued the mapping in the regions of Senegal River basin. Following this mapping, two MDA campaigns with praziquantel and albendazole were conducted in 2009 and 2010 in 14 health districts. These campaigns were only focused on SAC and adults at risk. In 2012, with the support from USAID, the Schistosomiasis Control Programme has conducted the mapping of regions located in the center and south of Senegal. The third MDA campaign was implemented in 2014 after complementary disease mapping conducted in 2013. All districts were treated regardless the prevalence level [11, 12].

The treatment of all endemic districts was realized in 2016 in accordance with WHO recommendations. After 4–5 years MDA implementation, WHO recommends countries to conduct impact evaluation of MDA on schistosomiasis and helminthiasis prevalence [13].

During this evaluation, WHO protocol for impact evaluation was used and parasitological methods such as urine filtration and sedimentation (urinary schistosomiasis) and Kato Katz (intestinal schistosomiasis) were used.

The impact evaluation conducted in early 2016, after five rounds of MDA campaigns for schistosomiasis in 24 districts located in Senegal Basin River, showed high prevalence of urinary schistosomiasis (54–96%) in 21 districts and low to moderate prevalence for intestinal schistosomiasis (0–33%) in 3 districts [14]. The evaluation performed in 2017 in 8 districts showed a prevalence between 0 and 69% in the central zone and a prevalence between 0 and 8% in the southern zone for *S. haematobium*. *S mansoni* was not observed in all sites [15]. Six districts were assessed in 2018 (four in the south and two in the center). In 2019, 3 districts in Thiès region and 5 districts in Senegal Basin River were evaluated [16].

Despite, MDA campaigns, urogenital schistosomiasis remains still endemic with a decrease of the prevalence while *S. mansoni* prevalence becomes very low [17]. These evaluations were carried out by the MoH in collaboration with the Department of Parasitology (UCAD), RTI ENVISION and OMVS.

According to WHO recommendation, the focal treatment was applied in 29 health posts located in areas with low schistosomiasis transmission in December 2019. The 2020 goals of reaching 75% of SAC was achieved in 27 health posts but 2 health posts located in Casamance (south part) did not reach this objective.

To eliminate schistosomiasis as public health concern, it’s become relevant to update the endemicity at community level in order to better target population requiring preventive chemotherapy with praziquantel. WHO in the new guidelines on control and elimination of human schistosomiasis recommends annual preventive chemotherapy with a single dose of praziquantel at 75% treatment coverage in all age groups from 2 years old including adults, pregnant women after the first trimester and lactating women, to control schistosomiasis morbidity and advance towards eliminating the disease as a public health problem [18].

This study was aimed to analyze data at community level in order to generate epidemiological data which could be used to update the endemicity of the disease for better targeting preventive chemotherapy with Praziquantel.

## Methods

### Sub-district description in Senegal

In Senegal, the operational level (district level) represents to the implementation level of health policies. The district level includes at least one health center and several health posts. The health center is managed by the District Chief Medical Officer who leads the district management team. The health post which represents community level, is headed by nurse and each health post has functional health huts managed by community health workers.

### Elaboration of the sub-district data workbook

In July 2019, the national team composed by NTD programme coordinator, the schistosomiasis programme manager and the data manager have participated in a training workshop for sub-district analysis and implementation in Brazzaville, Republic of Congo, organized by the Expanded Special Project for Elimination of NTDs (ESPEN), WHO/AFRO. During this meeting, demographic data (total population of the sub-district, percentage of SAC, percentage of adults, population of SAC, population of adults, year of population) and epidemiological data (number of people tested, number of positive tests, prevalence rate, methods of diagnostic, species tested, year of survey) from 77 districts and 1505 community health areas were presented.

A national review of schistosomiasis data was performed with the support of the Division of Research and Planification in Health (DPRS: Direction de la Planification et de la Recherche en Santé). This review allowed to add two new district and 43 sub-districts were added for a total 79 districts and 1548 community health areas. After this review, a briefing meeting with all NTDs team member, a quarterly coordination meeting of the NTD Program, a regional coordination meeting (11 regions) was organized. Emails containing all working documents and guideline were also sent to actors and partners.

In September 2019, a circular note from the MoH relating to the collection and transmission of schistosomiasis data was sent to all districts. In November 2019, 77 laboratory technicians were trained on schistosomiasis diagnosis. A training session on schistosomiasis guidelines was organized for regional health directors, health district officers, nurses, community health workers and teaching academy inspector in December 2019.

In September 2021, a national technical review meeting on schistosomiasis data was carried out. The purpose of this review was (i) to do a situational analysis of schistosomiasis control in Senegal; (ii) to update the endemicity of all health areas with the most appropriate data; (iii) to share results from operational research conducted in Senegal, (iv) to identify limits of implemented control strategies; (v) to develop a new roadmap for schistosomiasis control and elimination in Senegal; (vi) to analyze data at sub-district level in order to describe the health posts that should adjust their implementation strategy.

**Fig. 1:**
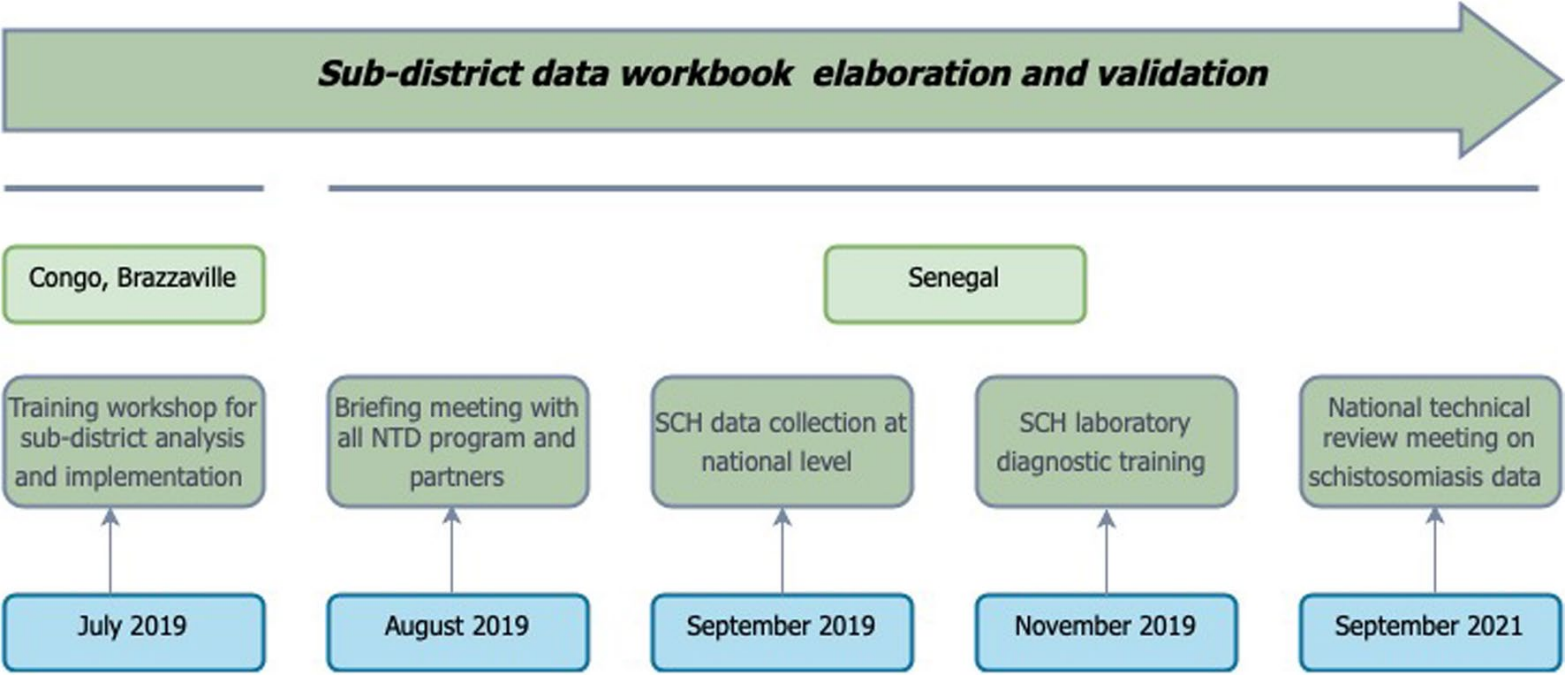
Sub-district data elaboration and validation

The methodology was based on presentation, review of scientific and epidemiological data, stakeholder discussions on the sub-district data and consensus building on intervention decisions in line with WHO guidelines. The presentations were mainly focused on an overview of the new NTD roadmap 2021–2030, the situation of schistosomiasis in Senegal, the disease mapping at sub-district level using WHO workbook and the results from studies conducted by research institutions and partner (Fig. 1).

### Sub-district data analysis tool and the use of decision in the country

The objective was to classify 1610 community health areas (CHA) according to the list provided by National Schistosomiasis Control Programme. This classification was made according to the risks of transmission of schistosomiasis into the following categories: (i) 0 = not endemic; (ii) 1 = low endemicity [< 10% by parasitological methods for intestinal and urogenital schistosomiasis or < 15% by circulating cathodic antigen (CCA) in *S. mansoni* endemic areas]; (iii) 2 = moderate endemicity (≥ 10% but < 50% by parasitological methods for intestinal and urogenital schistosomiasis or < 30% by questionnaire for history of hematuria or ≥ 15% but < 60% by CCA in *S. mansoni* endemic areas); and (iv) 3 = high endemicity (≥ 50% by parasitological methods for intestinal and urogenital schistosomiasis or ≥ 30% if based on questionnaires for history of hematuria or ≥ 60% by CCA in *S. mansoni* endemic areas) (https://espen.afro.who.int/system/files/content/resources/Schistosomiasis%20Data%20analysis%20tool%20-%20User%20Guide%20%28V1_20190916_English%29.pdf) [19].

Prior the classification, we reviewed the list of health post by district using data from demographic and environmental questionary, National Agency of Statistic and Demography and data from DHIS2. Population data of the health posts have been updated “demo data”. Epidemiological data was completed using results from operational research (UCAD, IRD, Espoir Santé, UGB/Saint Louis, UFR/Thiès) and investigation report. We also used the JRSM 2021, internet (Google Map), and telephone. The endemicity category was calculated at district level and community level using the decision tree proposed by WHO/ESPEN (Expended Special Project for Elimination of Neglected tropical Diseases), was also used to classify health areas for schistosomiasis endemicity [19].

### Quality control assessment

To finalize the data validation process at community level, a technical review was organized by the MoH in September 2021. All stakeholders working in the field of schistosomiasis were invited to participate in this activity. The Ministry was represented by the NTDs Coordinator, Schistosomiasis Programme Manager and other NTDs team members. The partners came from National Research Institutions (UCAD, UGB, UFR Santé/ Thies, IDR, Projet Espoir), ESPEN WHO (Consultant), USAID, FHI 360, Sightsavers, Enda Santé, OMVS, World Vision, Speak Up Africa. During this meeting, the technical working groups reviewed data collection tools, data from basic survey and impact evaluation, data from operational research. The endemicity criteria for the classification of health facilities and the criteria for mass treatment application were also reviewed. The sub-district workbook was further reviewed for correct naming of health areas, repetitions.

### Data analysis and statistical comparison

The WHO/AFRO sub-district workbook was used to analyze data from 1610 community health areas. The endemicity categories was determined using the WHO/ESPEN decision tree, WHO/AFRO sub-district data workbook and WHO/AFRO schistosomiasis risk assessment tool. The analysis was performed using Excel software. Descriptive analysis was performed. Quantitative variables were described in terms of means, standard deviation. For qualitative data, a description in terms of frequency with 95% confidence interval was used. Significance level of different tests was set at 5% two sides.

The main indicators calculated were: (i) number of community health areas according to the endemicity categories by region, (ii) the changes in sub-unit endemicity categories from implementation unit (IU)-level implementation; (iii) Endemicity at community level and population requiring treatment; (iv) the treatment adequacy according to the endemicity category; (v) the treatment adequacy according to the target population.

In application to the new WHO guidelines on control and elimination of human schistosomiasis a treatment strategy targeting all age groups from 2 years old including adults, pregnant women after the first trimester and lactating women have been determined as follow: “No endemicity,” “< 10% [no preventive chemotherapy (PC)]” and “≥ 10% (annual treatment)”.

## Results

### Demography: regions; districts, communities (CHA), and population

Overall, 1610 community health areas from 79 districts belongs to 14 regions have been analyzed. The districts cover a population of 17,156,624 habitants. SAC and adults’ populations represent 4,855,303 (28.3%) and 9,041,556 (52.7%) respectively.

**Fig. 2:**
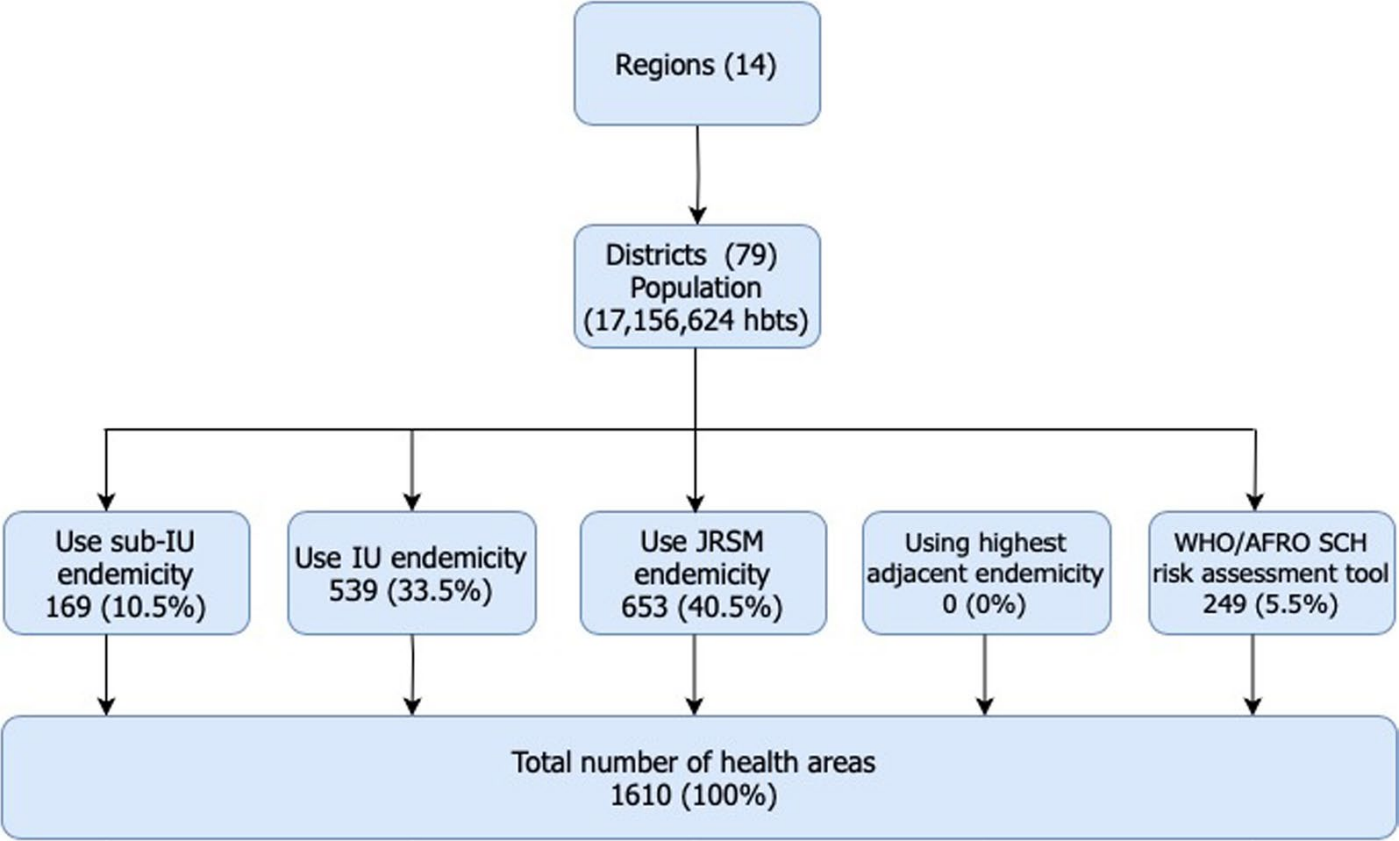
Different data sources used for disaggregation at community level. *hbts* habitants

1610 community implementation units (IUs) were categorized using the decision tree for schistosomiasis endemicity at district and community level (Fig. 2).

Evaluation of schistosomiasis risk transmission and the determination of endemicity categories were mainly based on the use of most recent JRSM endemicity data (40.5%) followed the use of district-IU endemicity (33.5%). Community-IU endemicity was in 10.5%. Highest adjacent endemicity was not used. According to the regions, same tendency was observed (Table 1).

**Table 1:** Sources of data for endemicity categories by regions

Region	Using Community endemicity	Using District endemicity	Using JRSM endemicity	Using highest adjacent endemicity	To be investigate	WHO/AFRO risk assessment tool	Total
Dakar	19	70	65	0	0	0	154
Diourbel	1	7	17	0	0	91	116
Fatick	19	36	25	0	0	49	129
Kaffrine	17	43	0	0	0	39	99
Kaolack	11	68	15	0	0	26	120
Kedougou	1	20	22	0	0	0	43
Kolda	6	42	25	0	0	0	73
Louga	3	24	100	0	0	1	128
Matam	20	10	74	0	0	0	104
Saint-Louis	9	44	78	0	0	0	131
Sedhiou	11	53	0	0	0	1	65
Tambacounda	8	18	114	0	0	0	140
Thiès	32	40	64	0	0	42	178
Ziguinchor	12	64	54	0	0	0	130
Total	169	539	653	0	0	249	1610
%	10.5	33.5	40.6	0.0	0.0	15.5	100.0

### Number of community health areas according to the endemicity categories by region

The results showed that 41.1% of community IUs (*n* = 666) have been classified as non-endemic. 282 (17.5%) and 398 (24.7%) community IUs have been classified as moderate and high endemicity respectively.

According to regions, high endemicity communities were in Tambacounda (*n* = 114), Saint Louis (*n* = 97), Matam (*n* = 78), Louga (*n* = 57) and Kedougou (*n* = 43) whereas the highest number of non-endemic areas were found in Thies (*n* = 153), Dakar (*n* = 152), Fatick (*n* = 96), Diourbel (*n* = 91) and Sedhiou (*n* = 44). Low endemicity was also found in 264 communities (16.4%). It was more important in Kaolack (*n* = 61) and Louga (*n* = 56) (Table 2).

**Table 2:** Number of community health areas (CHA) and targeted population according to endemicity categories by regions (*n* = 1610)

Regions	Total of communities	Total population	Non endemic			Low			Moderate			High		
			Number of CHA	Number of communities	Number of adults	Number of communities	Number of SAC	Number of adults	Number of communities	Number of SAC	Number of adults	Number of communities	Number of SAC	Number of adults
Dakar	154	18,943	152 (98.7)	0	0	2 (1.3)	18,943 (100)	0	0	0	0	0	0	0
Diourbel	116	157,211	91 (78.4)	0	0	4 (3.4)	51,334 (32.7)	0	21 (18.1)	77,145 (49.1)	28,732 (18.3)	0	0	0
Fatick	129	124,565	96 (74.4)	0	0	6 (4.7)	15,557 (12.5)	0	23 (17.8)	47,572 (38.2)	17,721 (14.2)	4 (3.1)	15,274 (12.3)	28,441 (22.8)
Kaffrine	99	111,142	48 (48.5)	0	0	32 (32.3)	58,955 (53.0)	0	19 (19.2)	38,026 (34.2)	14,161 (12.7)	0	0	0
Kaolack	120	311,751	27 (22.5)	0	0	61 (50.8)	198,804 (63.8)	0	31 (25.8)	73,422 (23.6)	27,344 (8.8)	1 (0.8)	4256 (1.4)	7925 (2.5)
Kedougou	43	158,479	0	0	0	0	0	0	0	0	0	43 (100)	55,371 (34.9)	103,108 (65.1)
Kolda	73	301,242	1 (1.4)	0	0	29 (39.7)	84,678 (28.1)	0	42 (57.5)	147,665 (49.0)	54,992 (18.3)	1 (1.4)	4859 (1.6)	9048 (3.0)
Louga	128	558,567	1 (0.8)	0	0	56 (43.8)	137,973 (24.7)	0	14 (10.9)	37,408 (6.7)	13,932 (2.5)	57 (44.5)	129,012 (23.1)	240,242 (43.0)
Matam	104	532,179	1 (1.0)	0	0	16 (15.4)	25,529 (4.8)	0	9 (8.7)	17,880 (3.4)	6660 (1.3)	78 (75.0)	168,440 (31.7)	313,670 (58.9)
Saint-Louis	131	822,705	0	0	0	0	0	0	34 (26.0)	57,377 (7.0)	21,371 (2.6)	97 (74.0)	259,924 (31.6)	484,033 (58.8)
Sedhiou	65	78,185	44 (67.7)	0	0	0	0	0	19 (29.2)	44,408 (56.8)	16,538 (21.2)	2 (3.1)	6023 (7.7)	11,216 (14.3)
Tambacounda	140	620,894	1 (0.7)	0	0	24 (17.1)	51,392 (8.3)	0	1 (0.7)	1,365 (0.2)	508 (0.1)	114 (81.4)	198,319 (31.9)	369,310 (59.5)
Thiès	178	14,289	153 (86.0)	0	0	9 (5.1)	44,987 (31.5)	0	15 (8.4)	61,911 (43.3)	23,060 (16.1)	1 (0.6)	4518 (3.2)	8414 (5.9)
Ziguinchor	130	121,403	51 (39.2)	0	0	25 (19.2)	32,105 (26.4)	0	54 (41.5)	65,067 (53.6)	24,231 (20.0)	0	0	0
Total	1,61	4,060,156	666 (41.4)	0	0	264 (16.4)	720,257 (17.7)	0	282 (17.5)	669,246 (16.5)	249,250 (6.1)	398 (24.7)	845,996 (20.8)	1,575,407 (38.8)

*SAC* school aged children

In high endemic settings, the proportion of SAC and adults represents 20.8% and 38.8% respectively. In moderate endemicity settings, the proportion of SAC and adults was 16.5% and 6.1% respectively. In low endemicity, only school aged children were concerned with a total number of 720, 257 (17.7%) (Table 4).

According to regions, Saint Louis, Matam and Louga have the highest number of SAC and adults exposed to schistosomiasis. In moderate endemic regions, the number of SAC exposed was more important in Ziguinchor (53.6%), Sédhiou (56.8%), Kolda (49.0%) and Diourbel (49.1%). In low endemic areas, only SAC were exposed (Table 2).

### Changes in community endemicity categories from district IU to community level implementation

**Fig. 3:**
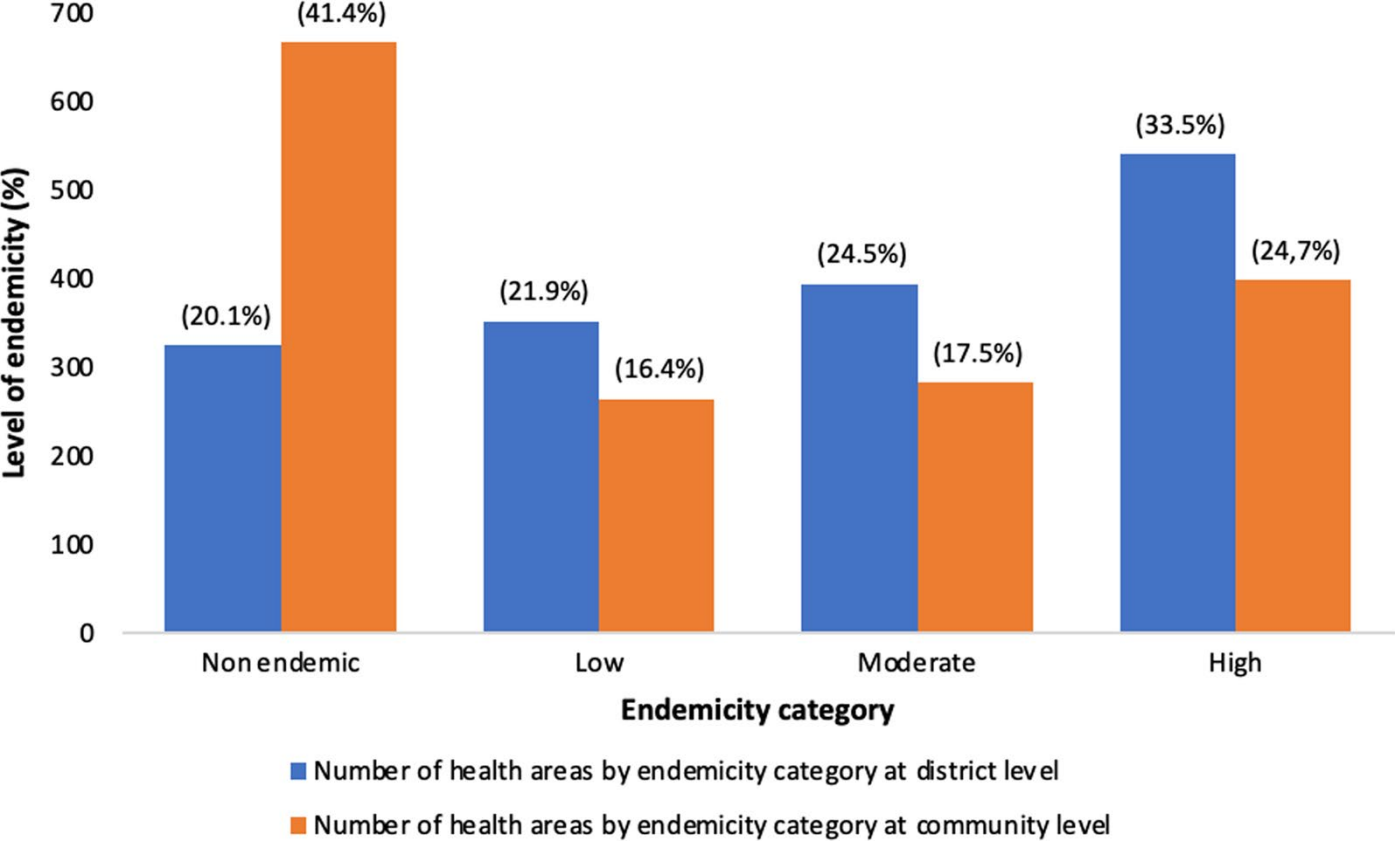
Comparison of endemicity categories from district level implementation to community level implementation (*N* = 1610)

The Fig. 2 illustrates the changes in endemicity category from district level to community level. At district level, 324 IUs (20.1%) were classified non endemic. When analyzing at community level, the number of non-endemic IU was almost twice more important (*n* = 666) (41.4%). Number of low endemic IU was high at district level was 352 (21.9%) while that at community level was 264 (16.4%). Same tendency was noted concerning high endemicity at district level compared to community level: 33.5% vs 24.7% (Fig. 3).

When looking the changes in endemicity category from district implementation level to community level implementation, the results have demonstrated that among 324 IUs classified as non-endemic at district level, 314 (96.9%) remain non endemics. However, 8 IUs (2.5%) considered non endemic become low at community level. In areas classified low endemic at district level (*n* = 352), 153 (43.5%) IUs remain low at community level. However, 186 (52.8%) and 13 (3.7%) become non endemic and moderate respectively. At district level, 394 IUs were classified moderate. The analysis at community level showed that only 176 (44.7%) remain moderate whereas 153 (38.8%), 60 (15.2%) and 5 (28.0%) IUs became respectively non endemic, low, and high. Among areas classified as high endemic at district level, 392 (72.6%) remain high, while 92 (17.0%) became moderate, 43 (8.0%) low and 13 (2.4%) non-endemic at community level (Table 3).

**Table 3:** Final endemicity category at community level

Endemicity category (Community level)	Number	Percentage (%)
Non endemic at district level implementation (*n* = 324)		
Non endemic	314	96.9
Low	8	2.5
Moderate	1	0.3
High	1	0.3
Low endemicity at district level implementation (*n* = 352)		
Non endemic	186	52.8
Low	153	43.5
Moderate	13	3.7
High	00	00
Moderate endemicity at district level implementation (*n *= 394)		
Non endemic	153	38.8
Low	60	15.2
Moderate	176	44.7
High	5	1.3
High endemicity at district level implementation (*n* = 540)		
Non endemic	13	2.4
Low	43	8
Moderate	92	17
High	392	72.6

### Community level endemicity and population requiring treatment

According to the target population and drug quantity, the number of areas requiring preventive chemotherapy is more important at district level (1286) compared to community level (944). The number of SAC requiring treatment is also more important at district level compared to community level. The difference between two levels is 144,850 SAC, representing (9.3%). This mean that less praziquantel drugs are required in community IU compared to districts IU (Table 4).

**Table 4:** Comparison of target population and drug quantities between district level implementation and community level implementation

	District level implementation	Community level implementation	Variation (%)
Number of sub-districts	1286	944	− 342 (26.6)
SAC requiring treatment	1,565,558	1,420,708	− 144,850 (9.3)
PZQ estimates	3,913,895	3,551,770	− 362,125 (9.3)

*SAC* school aged children,* PZQ* Praziquantel

### Treatment adequacy according to the endemicity category

Concerning the under treatment, at district level 10 IUs were classified non endemic (not requiring treatment). When analyzing at community level, the results showed that 8 IUs became low endemic, one became moderate and one high. IUs classified low and moderate at district level, became moderate and high at community level.

When regarding areas over treated, the results showed that areas classified moderate (213 IUs) at district level were not moderate at community level. 153 IUs (71.8%) were non endemic, and 60 IUs (28.2%) were low. Similar result was observed in high endemicity. Among 148 IUs classified high endemic, 13 IUs (8.8%) were non endemic, 43 IUs (29%) low and 92 IUs (62.2%) were moderate (Table 5).

**Table 5:** Treatment adequacy for community health areas by endemicity category, *n* (%)

PC strategy adequacy	District level implementation		Community level implementation			
			Community level endemicity category			
			Non endemic	Low	Moderate	High
Adequat treatment	Non endemic	314	314 (100%)	0	0	0
	Low	153	0	153 (100%)	0	0
	Moderate	176	0	0	176 (100%)	0
	High	392	0	0	0	392 (100%)
	Total	1035	314	153	176	392
Under treatment	Non endemic	10	0	8	1	1
	Low	13	0	0	13 (100%)	0
	Moderate	5	0	0	0	5 (100%)
	High	0	0	0	0	0
	Total	28	0	8 (28.6%)	14 (50%)	6 (21.4%)
Over treatment	Non endemic	0	0	0	0	0
	Low	186	186 (100%)	0	0	0
	Moderate	213	153 (71.8%)	60 (28.2%)	0	0
	High	148	13 (8.8%)	43 (29%)	92 (62.2%)	0
	Total	547	352	103	92	0
Total		1610	666	264	282	398

### Treatment adequacy according to the target population

Table 6 shows the treatment adequacy at district level and community level. Concerning the under treatment, the results showed that among 52,228 targeted population considered as non-endemic at district level, 43,143 (82.6%) remain non endemic but 4529 (8.7%) and 4556 (8.7%) became moderate and high respectively at community level. However, population considered low and moderate at district level keep same status at community level. For 282,869 people considered at high risk at district level, none of them was at high risk at community level. 30.2% were classified low and 69.8% were considered as moderate (Table 6).

**Table 6:** Treatment adequacy for target populations by endemicity category, *n* (%)

PC strategy adequacy	District level implementation		Community level implementation			
			Community level endemicity category			
			Non endemic	Low	Moderate	High
Adequat treatment	Non endemic	0	0	0	0	0
	Low	446,631	0	446,631 (100%)	0	0
	Moderate	443,141	0	0	443,141 (100%)	0
	High	821,910	0	0	0	821,910 (100%)
	Total	1,711,682	0	446,631	443,141	821,910
Under treatment	Non endemic	52,228	0	43,143 (82.6%)	4529 (8.7%)	4556 (8.7%)
	Low	24,200	0	0	24,200 (100%)	0
	Moderate	19,530	0	0	0	19,530 (100%)
	High	0	0	0	0	0
	Total	95,958	0	43,143	28,729	24,086
Over treatment	Non endemic	0	0	0	0	0
	Low	0	0	0	0	0
	Moderate	144,990	0	144,990 (100%)	0	0
	High	282,869	0	85,493 (30.2%)	197,376 (69.8%)	0
	Total	427,859	0	230,483	197,376	0
Total		2,235,499	0	720,257	669,246	845,996

### School aged children adequatly treated according to the regions

According to regions, SAC living in Louga, Saint Louis and Kaolack were more treated adequately with 15.6%, 15.2% and 14.7% respectively. Thiès, Kaolack and Dakar have the highest proportion of SAC under treated. Over treatment was highly found in Kolda (28.9%) and Kaffrine (13.8%).

The quantity of drugs underestimated was more important in Thiès (30.8%), Fatick (21.9%), Dakar (17.9%) and Kaolack (16.4%). Drugs overestimated were more frequent in Kolda (20.4%) and Tambacounda (19.4%) (Table 7).

**Table 7:** Treatment adequacy according to the regions

Regions	Number of communities (%)	SAC adequatly treated, *n* (%)	SAC under treated, *n* (%)	SAC over treated, *n* (%)	Under estimated drugs, *n* (%)	Over estimated drugs, *n* (%)
Dakar	154 (9.6)	0 (0.0)	18,943 (19.7)	0 (0.0)	15,628 (17.9)	0 (0.0)
Diourbel	116 (7.2)	128,479 (7.5)	0 (0.0)	0 (0.0)	0 (0.0)	0 (0.0)
Fatick	129 (8.0)	63,129 (3.7)	15,274 (15.9)	0 (0.0)	19,092 (21.9)	0 (0.0)
Kaffrine	99 (6.1)	38,026 (2.2)	0 (0.0)	58,955 (13.8)	0 (0.0)	25,054 (5.5)
Kaolack	120 (7.5)	250,955 (14.7)	25,527 (26.6)	0 (0.0)	14,361 (16.4)	0 (0.0)
Kedougou	43 (2.7)	55,371 (3.2)	0 (0.0)	0 (0.0)	0 (0.0)	0 (0.0)
Kolda	73 (4.5)	113,586 (6.6)	0 (0.0)	123,616 (28.9)	0 (0.0)	92,163 (20.4)
Louga	128 (8.0)	266,985 (15.6)	0 (0.0)	37,408 (8.7)	0 (0.0)	46,760 (10.4)
Matam	104 (6.5)	168,440 (9.8)	0 (0.0)	43,409 (10.1)	0 (0.0)	65,111 (14.4)
Saint-Louis	131 (8.1)	259,924 (15.2)	0 (0.0)	57,377 (13.4)	0 (0.0)	71,718 (15.9)
Sedhiou	65 (4.0)	1467 (0.1)	4556 (4.7)	44,408 (10.4)	11,390 (13)	55,510 (12.3)
Tambacounda	140 (8.7)	198,319 (11.6)	0 (0.0)	52,757 (12.3)	0 (0.0)	87,786 (19.4)
Thies	178 (11.1)	69,829 (4.1)	31,658 (33)	9929 (2.3)	26,871 (30.8)	7433 (1.6)
Ziguinchor	130 (8.1)	97,172 (5.7)	0 (0.0)	0 (0.0)	0 (0.0)	0
Total	1610 (100)	1,711,682 (100)	95,958	427,859 (100)	87,342 (100)	451,535 (100)

*SAC* school aged children

According to the new WHO guidelines on control and elimination of human schistosomiasis (2022), the results (see Table 8) showed that the annual treatment with praziquantel concerned only population living in endemic areas with the prevalence ≥ 10%. It concerns 680 (42.2%) of communities with a total of 1,515,242 (34.9%) SAC and 2,821,682 (65.1%) adults. The mains regions with community health areas requiring the annual treatment are Saint Louis, Louga, Matam, Tambacouda, Kédougou and Kolda (Table 8).

**Table 8:** Number of communities health areas by treatment regime and target population

Region	Total of communities	Total population	Non endemicity			< 10% (Pas de CP)			≥ 10% (1 tour/an)		
			Number of communities (%)	Number of SAC	Number of adults	Number of communities (%)	Number of SAC	Number of adults	Number of communities (%)	Number of SAC (%)	Number of adults (%)
Dakar	154	0	152 (98.7)	0	0	2 (1.3)	0	0	0	0	0
Diourbel	116	220,806	91 (78.4)	0	0	4 (3.4)	0	0	21 (18.1)	77,145 (34.9)	143,661 (65.1)
Fatick	129	179,878	96 (74.4)	0	0	6 (4.7)	0	0	27 (20.9)	62,846 (34.9)	117,032 (65.1)
Kaffrine	99	108,840	48 (48.5)	0	0	32 (32.3)	0	0	19 (19.2)	38,026 (34.9)	70,814 (65.1)
Kaolack	120	222,332	27 (22.5)	0	0	61 (50.8)	0	0	32 (26.7)	77,678 (34.9)	144,654 (65.1)
Kedougou	43	158,479	0	0	0	0	0	0	43 (100)	55,371 (34.9)	103,108 (65.1)
Kolda	73	436,549	1 (1.4)	0	0	29 (39.7)	0	0	43 (58.9)	152,524 (34.9)	284,025 (65.1)
Louga	128	476,322	1 (0.8)	0	0	56 (43.8)	0	0	71 (55.5)	166,420 (34.9)	309,902 (65.1)
Matam	104	533,288	1 (1.0)	0	0	16 (15.4)	0	0	87 (83.7)	186,320 (34.9)	346,968 (65.1)
Saint-Louis	131	908,186	0	0	0	0	0	0	131 (100)	317,301 (34.9)	590,885 (65.1)
Sedhiou	65	144,340	44 (67.7)	0	0	0	0	0	21 (32.3)	50,431 (34.9)	93,909 (65.1)
Tambacounda	140	571,536	1 (0.7)	0	0	24 (17.1)	0	0	115 (82.1)	199,684 (34.9)	371,852 (65.1)
Thiès	178	190,134	153 (86.0)	0	0	9 (5.1)	0	0	16 (9.0)	66,429 (34.9)	123,705 (65.1)
Ziguinchor	130	186,234	51 (39.2)	0	0	25 (19.2)	0	0	54 (41.5)	65,067 (34.9)	121,167 (65.1)
Total	1610	4,336,924	666 (41.4)	0	0	264 (16.4)	0	0	680 (42.2)	1,515,242 (34.9)	2,821,682 (65.1)

*SAC* school aged children

## Discussion

### Endemicity and the use of decision tree

This analysis was conducted for updating the endemicity of schistosomiasis at community level for better targeting population requiring preventive with praziquantel. The results from the analysis show that 40.5% and 33.5% of data from the decision tree was generated using JRSM and the district endemicity. The community endemicity was used in 10.5%. The WHO/AFRO risk transmission assessment tool was also used to update endemicity in 5.5%. The results demonstrated that the JRSM form, the district-endemicity, the community-endemicity and the WHO/AFRO risk transmission assessment tool could be considered as a useful tool for updating the endemicity of schistosomiasis. However, the interpretation and validation of the data require a large concertation with the participation of all stakeholders (schistosomiasis programme, operational level, scientists, epidemiologists, parasitologists, community health workers, water and sanitation specialists and partners).

### Endemicity

According to regions, the results showed that high endemicity communities are located in Tambacounda, Saint Louis, Matam, Louga and Kedougou. These regions represent the Senegal River Bassin. Since the construction of Diama Dame, schistosomiasis became endemic in these areas. This was demonstrated by Southgate et al. in 1997 when assessing the effects after the construction of the Dams of Diama and Manantali [20]. Piquet et al. have found similar results [21]. In 2009 Meurs et al. found high prevalence of *S. mansoni* (61%) and *S. haematobium* (50%) in the north of Senegal [4]. A recent impact evaluation conducted by the SCH/STH Programme in Senegal River Bassin have showed that schistosomiasis is still endemic in this arear with prevalence ranging from 18% to 96% in the Delta and from 3% to 93% in the river valley [14]. Similar results were described previously [12]. The results from our study were confirmed by Ndiaye et al. when evaluation the impact of Schistosomiasis Control Program in Ethnic group in Kedougou [22].

The persistence of high endemicity in these regions is due to the location of these regions along the Senegal Bassin River and the frequent contact of population with water due their domestic activities.

Highest number of non-endemic communities were found in Thies and Dakar. These communities are known as non-endemic areas for schistosomiasis. However, in Factick and Sedhiou, schistosomiasis is noted in some districts. In Niakhar district located in Fatick region, one previous study on the protective effect of schistosomiasis against malaria was carried out in two villages (Tukar and Diokhine), and the overall prevalence of urinary schistosomiasis was 67% [23]. Same tendency was observed by Senghor et al. when assessing the prevalence and intensity of urinary schistosmiasis in school children [24].

More than 60% of IUs in Sedhiou are non-endemics but 35% are classified moderate and high endemicity. Similar results were previously described by the Schistosomiasis Control Program [25].

Low endemicity was also found in 264 communities and it was more important in Kaolack and Louga. These regions are known as areas of low endemicity because of temporary transmission.

Recent studies showed that the prevalences varied from 10% in the central regions with seasonal transmission, to over 95% in the Senegal River Basin where the transmission is perennial [26, 27].

### Changes in endemicity categories

Results from the analysis have demonstrated that there is a considerable change in endemicity category from district implementation level to community implementation level. The number of non-endemic IUs was more important at community level compared to district level. A decrease of IU classified high endemic was noted from district level to community level. The same tendency was also observed concerning the low and moderate endemicity.

Similar results were noted concerning the population exposed. Population number decrease from district to community level. These results suggest, there is better classification and better target of population exposed.

Based on these result, better planning of preventive chemotherapy campaign could be done to optimize the use of praziquantel at community level. If PC is well planned, it will allow to reduce morbidity related to schistosomiasis. This was demonstrated by Senghor et al. when evaluation the impact of annual praziquantel treatment on urogenital schistosomiasis in central part of Senegal from 2011 to 2014. A significance decrease of the prevalence was noted (from 57.7% to 10.1%) [9]. When assessing the effect of preventive chemotherapy with praziquantel on schistosomiasis among school aged children in sub-Saharan Africa, Kokaliaris et al.’s research showed that preventive chemotherapy with praziquantel can decrease considerably the prevalence of schistosomiasis. They showed that *Schistosoma* prevalence among school aged children decreased from 23% in 2000–2010 to 9.6% in 2015–2019, an overall reduction of 58.3% [28].

### Treatment accuracy

The community data analysis has demonstrated that PC implementation strategy at district IU level is subject to “under treatment” and “over treatment” both in the number of community health areas (26.6% reduction) as for the number of target population requiring treatment (9.3% reduction). The results showed that among 10 IUs classified non endemic at district level, 8 IUs became low endemic, one became moderate and one high. IUs classified low and moderate at district level, became moderate and high at community level respectively. These results suggest that the 20 IUs were under treated at district level. In terms of population, 52,815 habitants requiring preventive chemotherapy were not treated. This part of the population continues to be potential sources of diseases transmission. When mass drug administration is well planned, the impact must be a reduction of disease transmission. This has been demonstrated by Stothard et al. who showed a relative reduction of schistosomiasis prevalence of 47.5% (2011–2014) and 64.5% (2015–2019) [29]. When regarding the over treatment, among areas classified moderate (213 IUs) at district level, 153 IUs (71.8%) were non endemic, and 60 IUs (28.2%) were low at community level. Similar result was observed in high endemicity, among 148 IUs classified high endemic, 13 IUs (8.8%) were non endemic, 43 IUs (29.0%) low and 92 IUs (62.2%) were moderate. The consequences are important in terms of drugs use and resources deployed. All these districts usually planned for annual MDA are no longer necessary which has a significant impact in saving resources, logistic and drugs. The planned resources could be adequately relocated to community level where appropriate.

Overall, under-treatment and over-treatment have been observed with implication on drug estimation and number of populations requiring PC according to WHO guidelines.

The elimination schistosomiasis as a public health problem requires a better targeting of populations requiring preventive chemotherapy and by considering all populations at risk including pre-school aged children (pre-SAC), school aged children and all adult at risk. Concerning the pre-SAC living in high endemic areas with high intensity of infection, the development of pediatric formulation could allow to cover this age group so far excluded in mass drug administration campaign. Several studies have shown the effectiveness of pediatric formulation [30, 31].

This study presented several limitations. The inadequate data to inform the analysis tool, the lack of risk assessment data to inform outcomes of the tool, and the limitations and gaps in some cases of the local knowledge for each community, by the MoH staff participating in data validation could be considered as limitations of the study.

## Conclusions

The results of this study showed that analysis at community level could allow for better optimization of the use of praziquantel at the community level. This analysis at community level through the WHO decision tree allows to update the endemicity of schistosomiasis and to better targeting the populations requiring preventive chemotherapy. By allowing to avoid under- or over-treating populations, this concept of analysis at community level could be of capital help in achieving the objectives set by the WHO.

## Data Availability

Data used in this analyses is routine programmatic data in use by the MoH of Senegal, and shared with the World Health Organisation. The MoH Senegal signed consent with WHO /AFRO in 2017 to allow for sharing of PC-NTD programme data on the ESPEN portal https://espen.who.int.

## Abbreviations

CCA: Circulating cathodic antigen
CHA: Community health areas
CMS: Division du Contrôle Médicale Scolaire
DLM: Direction de la Lutte contre la Maladie
DPRS: Direction de la Planification et de la Recherche en Santé
ESPEN: Expanded Special Project for Elimination of Neglected Tropical Diseases
IU: Implementation unit
IRD: Institute of Research and Development
JRSM: Join request for Selected PC Medicine
MDA: Mass drug administration
MSAS: Ministère de la Santé et de l’Action sociale
NTD: Neglected tropical diseases
OMVS: Organisation pour la Mise en Valeur du fleuve Sénégal
PC: Preventive chemotherapy
PNLB: Programme National de Lutte contre la Bilharziose
PZQ: Praziquantel
SAC: School aged children
SCH: Schistosomiasis
UCAD: University Cheikh Anta Diop
UFR: Unité de Formation et de Recherche
UGB: University Gaston Berger
WHO: World Health Organization

## References

[1] French MD, Evans D, Fleming FM, Secor WE, Biritwum N-K, Brooker SJ (2018). Schistosomiasis in Africa: improving strategies for long-term and sustainable morbidity control. PLoS Negl Trop Dis.

[2] WHO. Schistosomiasis and soil-transmitted helminthiases: number of people treated in 2016. Wkly Epidemiol Rec. 201;92(49):749–60. 29218962

[3] Talla I, Kongs A, Verlé P (1992). Preliminary study of the prevalence of human schistosomiasis in Richard-Toll (the Senegal river basin). Trans R Soc Trop Med Hyg.

[4] Meurs L, Mbow M, Vereecken K, Menten J, Mboup S, Polman K (2012). Epidemiology of mixed *Schistosoma mansoni* and *Schistosoma haematobium* infections in northern Senegal. Int J Parasitol.

[5] Ernould JC. Épidémiologie des schistosomoses humaines dans le delta du fleuve Sénégal: phénomène récent de compétition entre *Schistosoma haematobium* Sambon, 1907 et *S. mansoni* (Bilharz, 1852) [PhD thesis]. Université de Paris 12: Val de Marne, Médecine Parasitologie, 1996, 602 p.

[6] Diaw OT, Seye M, Sarr Y (1988). Épidémiologie des trématodoses du bétail dans la région de Kolda, Casamance Sénégal. Rev El Méd Vét Pays Trop.

[7] Sy I, Diawara L, Ngabo D, Barbier D, Dreyfuss G, Georges P (2008). Schistosomiasis in school children in the Bandafassi region of East Senegal. Med Trop.

[8] Chippaux JP. La lutte contre les schistosomoses en Afrique de l’Ouest. IRD Editions; 2000, 225–236.

[9] Senghor B, Diaw OT, Doucoure S, Seye M, Diallo A, Talla I, Bâ CT, Sokhna C (2016). Impact of annual praziquantel treatment on urogenital schistosomiasis in a seasonal transmission focus in Central Senegal. PLoS Negl Trop Dis.

[10] Ministère de la santé, Sénégal. Plan stratégique de Lutte Intégrée contre les Maladies Tropicales Négligées 2016–2020

[11] Ministère de la santé, Sénégal. Plan stratégique de Lutte Intégrée contre les Maladies Tropicales Négligées 2007–2011

[12] Ministère de la santé, Sénégal. Plan stratégique de Lutte Intégrée contre les Maladies Tropicales Négligées 2011–2015

[13] World Health Organization. Helminth control in school-age children: a guide for managers of control programmes. World Health Organization; 2011. 2nd edition. 2011. https://apps.who.int/iris/bitstream/handle/10665/44671/?sequence=1.

[14] Ministère de la Santé et de l’Action Sociale, Sénégal / USAID / RTI ENVISION. Résultats de l’évaluation de l’impact des traitements effectues contre les schistosomiases et les géohelminthiases dans le bassin du fleuve Sénégal. Rapport 2016.

[15] Ministère de la Santé et de l’Action Sociale, Sénégal / USAID /RTI ENVISION. Rapport scientifique. Evaluation de l’impact des traitements effectués contre les schistosomiases et les géohelminthiases. Rapport 2017.

[16] Ministère de la Santé et de l’Action Sociale, Sénégal / USAID /RTI ENVISION. Rapport de l’évaluation de la prévalence de la bilharziose et des géohelminthiases dans les districts de Medina yoro foulah, Kaffrine, Koungheul, Goudomp, Sedhiou et Bounkiling. Rapport 2018.

[17] OMVS (Organisation pour la mise en Valeur du fleuve du Sénégal). (2020) Rapport scientifique: Etudes de base de la schistosomiase dans les sites sentinelles du bassin du fleuve Sénégal. *Rapport d’Etude* 2020.

[18] World Health Organization. WHO guideline on control and elimination of human schistosomiasis. World Health Organization; 2022 Feb 15. ISBN: 978 92 4 0041608. Geneva. https://www.who.int/publications/i/item/9789240041608. 35235279

[19] World Health Organization. Optimizing Schistosomiasis MDA Implementation: Sub-Implementation Data Analysis Tool, User Guide Version 1.0. 2019. p 17–21. https://espen.afro.who.int/system/files/content/resources/Schistosomiasis%20Data%20analysis%20tool%20-%20User%20Guide%20%28V1_20190916_English%29.pdf.

[20] Southgate VR (1997). Schistosomiasis in the Senegal River Basin: before and after the construction of the dams at Diama, Senegal and Manantali, Mali and future prospects. J Helminthol.

[21] Picquet M, Ernould JC, Vercruysse J, Southgate VR, Mbaye A, Sambou B (1996). Royal Society of Tropical Medicine and Hygiene meeting at Manson House, London, 18 May 1995. The epidemiology of human schistosomiasis in the Senegal river basin. Trans R Soc Trop Med Hyg.

[22] Léger E, Borlase A, Fall CB, Diouf ND, Diop SD, Yasenev L (2020). Prevalence and distribution of schistosomiasis in human, livestock, and snail populations in northern Senegal: a One Health epidemiological study of a multi-host system. Lancet Planet Health.

[23] N'Diaye M, Dioukhane EM, Ndao B, Diedhiou K, Diawara L, Talla I (2016). Schistosomiasis sustained control program in ethnic groups around Ninefescha (Eastern Senegal). Am J Trop Med Hyg.

[24] Senghor B, Diallo A, Sylla SN, Doucouré S, Ndiath MO, Gaayeb L (2014). Prevalence and intensity of urinary schistosomiasis among school children in the district of Niakhar, region of Fatick, Senegal. Parasit Vectors.

[25] Briand V, Watier L, Le Hesran JY, Garcia A, Cot M (2005). Coinfection with *Plasmodium falciparum* and *Schistosoma haematobium*: protective effect of schistosomiasis on malaria in senegalese children?. Am J Trop Med Hyg.

[26] Global Atlas of Helminths Infection (GAHI). Distribution of *S. haematobium* survey data in Senegal. Global Atlas of Helminth Infections. 2012. https://www.thiswormyworld.org/maps/distribution-of-s-haematobium-survey-data-in-senegal.

[27] Webster BL, Diaw OT, Seye MM, Faye DS, Stothard JR, Sousa-Figueiredo JC, Rollinson D (2013). Praziquantel treatment of school children from single and mixed infection foci of intestinal and urogenital schistosomiasis along the Senegal River Basin: monitoring treatment success and re-infection patterns. Acta Trop.

[28] Kokaliaris C, Garba A, Matuska M, Bronzan RN, Colley DG, Dorkenoo AM (2022). Effect of preventive chemotherapy with praziquantel on schistosomiasis among school-aged children in sub-Saharan Africa: a spatiotemporal modelling study. Lancet Infect Dis.

[29] Stothard JR, Sousa-Figueiredo JC, Betson M, Green HK, Seto EY, Garba A (2011). Closing the praziquantel treatment gap: new steps in epidemiological monitoring and control of schistosomiasis in African infants and preschool-aged children. Parasitology.

[30] Ekpo UF, Oluwole AS, Abe EM, Etta HE, Olamiju F, Mafiana CF (2012). Schistosomiasis in infants and pre-school-aged children in sub-Saharan Africa: implication for control. Parasitology.

[31] Coulibaly JT, Panic G, Silué KD, Kovač J, Hattendorf J, Keiser J (2017). Efficacy and safety of praziquantel in preschool-aged and school-aged children infected with *Schistosoma mansoni*: a randomised controlled, parallel-group, dose-ranging, phase 2 trial. Lancet Glob Health.

