# Correction: Schistosomiasis control in Senegal: results from community data analysis for optimizing preventive chemotherapy intervention with praziquantel

**DOI:** 10.1186/s40249-024-01217-0

**Published:** 2024-06-25

**Authors:** Boubacar Diop, Khadime Sylla, NDèye MBacké Kane, Oumou Kaltoum Boh, Babacar Guèye, Mady Ba, Idrissa Talla, Malang Mané, Rose Monteil, Boniface Kinvi, Honorat Gustave Marie Zoure, Jorge Cano Ortega, Pauline Mwinzi, Moussa Sacko, Babacar Faye

**Affiliations:** 1 Ministère de La Santé Et de L’Action Sociale (MSAS), Programme National de Lutte Contre Les Bilharzioses Et Les Géo-Helminthiases, Direction de La Lutte Contre La Maladie (DLM), Dakar, Senegal; 2 Department of Parasitology and Mycology Faculty of Medicine, Pharmacy and Odontology, Université Cheikh Anta Diop de Dakar (UCAD), Dakar, Senegal; 3 WHO Country Office, Dakar, Senegal; 4 Département de Santé Publique, Faculté Des Sciences de La Santé, Université Amadou Hampathé Ba, Dakar, Senegal; 5 Chargé Des Enquêtes Et Evaluation Des Maladies Et Des Campagnes de DMM, FHI 360, Dakar, Senegal; 6 Expanded Special Project for Elimination of Neglected Tropical Diseases (ESPEN), WHO Africa Regional Office, Brazzaville, Congo; 7 Service de Parasitologie, Institut National de Santé Publique (INSP), BP 1771, Bamako, Mali

**Correction** **:** **Infect Dis Poverty 12, 106 (2023)**

**https://doi.org/10.1186/s40249-023-01155-3**

Following publication of the original article [1], the authors reported that they would like to add the following statement to the Acknowledgement:

We would like also to acknowledge the CEA AGIR (Centre d’Excellence Africain: Environnement, Santé, Sociétés) for supporting the publication of this manuscript.
